# Study on crack evolutional behavior of rocks in triaxial compression based on colony growth dynamics model

**DOI:** 10.1038/s41598-022-23177-x

**Published:** 2022-11-02

**Authors:** Naifu Deng, Lan Qiao, Qingwen Li, Jiawang Hao, Mengxi Wei, Qinglong Zhang

**Affiliations:** 1 State Key Laboratory of High-Efficient Mining and Safety of Metal Mines, University of Science and Technology Beijing, Beijing, 100083 China; 2 Beijing Key Laboratory of Urban Underground Space Engineering, University of Science and Technology Beijing, Beijing, 100083 China; 3 University of Science and Technology Beijing, Beijing, 100083 China

**Keywords:** Civil engineering, Petrology, Mineralogy

## Abstract

The crack propagation behavior of rock during compression involves complex mechanisms. Describing the growth behavior of a large number of cracks with conventional mechanical models is a major challenge. Therefore, in this work, we propose a new method to describe crack growth behavior by considering crack bodies as free voxels that can expand and coalesce within a rock sample according to certain rules. Specifically, we first propose a crack growth model that quantitatively describes the crack growth ratio and crack growth rate, which are integrally related to the loading rate, internal friction angle, cohesion, initial porosity, and confining stress. Second, to avoid the complex analytical process of the traditional mechanical model in solving the propagation directions of multiple cracks, we introduce a method for determining the crack growth directions of shearing failure based on the colony growth assumption. This method defines the crack propagation direction as a synthetic vector of the inertial direction, the attractive direction, the Coulomb direction, and the edge direction. Moreover, a new mathematical description method of fracture energy and plastic energy is proposed to calculate the crack growth at each time step. The simulation results show that our crack growth model for shearing failure agrees well with the experimental results and explains the fracture behavior and transformation law of cracks to some extent.

## Introduction

The evolution process of cracks within the rock, such as initiation, propagation and coalescence, within the rock has long been a research focus and difficult problem. Rock fragmentation caused by engineering disturbance under high confining stress poses a concern to downstream operations like excavation and haulage as a result of the rising need for deep excavating resources. Therefore, it is inevitable and essential to analyze fracture expansion patterns under high-stress situations. In actual underground works such as tunneling and mining, the surrounding rock is inherited with numerous tiny defects such as joints and faults. Due to the complex heterogeneous stress state in deep underground works, the surrounding rocks are prone to fracture due to its inherent defects, leading to severe engineering disasters such as rock stripping, ejection and zonal disintegration^2–4^. A variety of abnormal phenomena in deep surrounding rock have been observed due to the increasing exploration depth of the underground work. For instance, deep rocks possess higher crack expansion rate and energy storage and therefore are more prone to burst under excavated disturbance^5^. Furthermore, the damage mode of rock will transform from brittle to ductile when increasing the depth^6^. It is concluded that during excavation, fractures are prone to initiate from the primary cracks due to non-uniformity energy accumulation around the crack tips^7^. Additionally, rock failure in deep depth is closer to shearing failure pattern rather than splitting pattern because the high surrounding pressure environment compresses the existing fractures and causes the fractures to propagate in a direction more inclined to slip along the shear surface. Therefore, the study of shearing fracture behavior under deep surrounding rocks is of great value for the prevention and control of deep underground works.

Many research efforts have been made in seeking the intrinsic and fundamental laws of fracture through different research tools, such as Infrared radiation monitoring (IRR)^8^, acoustic emission (AE) method^9^, strain field monitoring^10^ and equivalent transparent material simulation^11^. As an effective non-contact monitoring method, infrared radiation detection has been widely used in the study of rock damage and fracture mechanisms^12,13^. Sun et al. in 2017 found that infrared thermography can classify the compression process of rock samples into three stages before failure, i.e., the compaction stage, the elastic stage and the plastic stage. Based on the three-stage signals obtained by infrared radiation, Jiawang et al. in 2021 conducted true triaxial compressive tests on preprocessed sandstones under infrared monitoring and concluded that high confining stresses promote the development of cracks from the initial crack tip to the horizontal direction^7^. Research has been conducted to verify the correlation between the AE signals and rock failure that the initiation, closure, propagation, coalescence of the crack are reflected as different typical AE amplitude and frequency-spectrum^14,15^. Scholars also found that the location and propagation path of cracks can be obtained from the AE signal sources, which indicates that the crack propagation path is relatively consistent with the Mohr Coulomb Criterion^16,17^, namely, the orientation of the fracture is consistent with the rupture angle. Strain-field monitoring is another frequently-used method normally utilizing Digital Image Correlation (DIC) to observe the evolution of cracks on the rock surface, which has been proven useful for deformation-related strain heterogeneity in rock masses^18,19^. IRR, AE method and strain field monitoring are non-contact ways to capture the indirect signals to analyze the fracture extension. However, these methods cannot visualize the process of the inner development of the cracks, thus, many scholars conducted experiments with equivalent transparent material such as polymethylmethacrylate and photopolymer, from which equivalent "rock samples" made with carefully designed cracks are embedded^11,20–22^, allowing a well observation of the crack evolution during the compressive test. However, due to the limitations of equipment, this method can only simulate the fracture behavior of macroscopic cracks by omitting the existence of microstructure.

In addition to the qualitative or descriptive research mentioned above for exploring the law of crack evolution, scholars have proposed many simulation models to quantitatively describe the state of porous rocks under loading such as the elastic spider network model^23^, cube structure model^24^ and Gibson–Ashby model^25^. These models are well known for simulating internal microstructures of porous materials, but they are relatively simple due to many assumptions made for simplification. With the help of various methods such as serial sectioning method^26^, confocal laser scanning microscopy method^27^, FIB-SEM method^28^ and micro-CT method^29^, scholars can easily acquire the real microstructures of the rocks, especially, the micro-CT and X-ray CT methods greatly promote the research work of reconstruction of real porous rocks. Based on the X-ray CT data, Zhou et al. proposed a mathematical model with spherical harmonics to reconstruct the porous microstructure of rocks^30^. Zhou et al. also utilized X-ray micro-CT technology to obtain the micro-structural morphological pattern of the cracks which was used to reconstruct the 3D tetrahedral model by using Delaunay refinement algorithm, then, the reconstruct model were verified by numerical simulation through transforming into a finite element model^31,32^. Although the above research work can precisely illustrate the static state of real microstructure of porous rocks, the evolution of cracks under loading is not included.

In recent years, very limited studies have considered mathematical models to explain or simulate crack propagation under loading, and most scholars prefer to use the finite element method (FEM) or discrete element method (DEM) for numerical simulation of crack development. Yan et al. built a coupled thermo-mechanical model based on a combined finite-discrete element method (FDEM) to simulate the thermal cracking of rocks. This model combined the heat conduction equation and fracture mechanism to perform prediction of the crack extension^33^. However, this study did not provide insight into the fracture propagation mechanism, but only analyzed the correlation between fracture growth and indirect thermal indices. Ehsan et al. conducted an in-depth study of the fracture mechanism of heterogeneous anisotropic rocks using the extended finite element method (XFEM), and the results showed that this formulation has great potential for estimating the stress intensity factor (SIF) and estimating the fracture extension trajectory^34^. Li et al. also proposed an optimized model using a grain-based finite-discrete element method (GB-FDEM) according to the calibration results of benchmark experiments including uniaxial compression test, confined compression test and Brazilian disc test, which focus on the fracture process of brittle rocks and the damage behavior due to crack initiation and propagation prior to peak stress^35^. The aforementioned studies provide a good theoretical basis for the analysis of crack evolution but currently, most related research results are based on FEM or DEM which is not a direct tool to simulate crack extension from the view of cracks.

In this paper, we proposed a novel crack growth model from the view of cracks seeking the colony behavior of fracture. The main contributions of this paper are summarized as follows:

1. The proposed crack growth model defines the ratio of cracks $C$ as the volumetric ratio of the fractured voxels transformed from rock skeletons and provide an analytical solution for $C$ based on the Mohr Coulomb Criterion.
2. Our model also defines the propagation direction of each crack as a resultant vector governed by four factors, namely, the inertial direction, the attractive direction, the Mohr Coulomb direction and the edge attraction direction.
3. Furthermore, the model divides the fracture behaviors under compression into four stages, that is the elastic stage, the crack initiation stage, the stable growth stage and the unstable growth stage, which is consistent with the results of previous studies.
4. A novel definition of the plastic energy and the fracture energy absorbed during compression is proposed accordingly. To analytically describe the increment of each crack, this paper also introduces the unit resistant energy for each crack which indicates that fracturing occurs as the fracture energy absorbed by the crack is greater than the unit resistant.
5. This paper proposes a novel way to compute the initial fracture stress and the damage stress based on the crack growth model, which provides enlightenment to know stress level at different compression stage by knowing some basic mechanical parameters.

## Methodology

Natural rocks are inherited with fractures and pores which are randomly distributed within the rocks. indoor experiments and simulated results find that new cracks will initiate from these natural defects (fractures and pores) in compression^36–38^. In details, the fracture morphology of rocks under compression tends to exhibit a cluster phenomenon, i.e., cracks tend to propagate and coalesce with each other to form a specific damage pattern. This fracture behavior is very similar to the growth behavior of most populations in nature. Usually, the growth of individuals in a population is closely related to the growth pattern of the population, and the individuals will interact, attract and integrate with each other, finally forming a specific community pattern. To verify whether the crack growth behavior can be explained by a similar theory, this paper presents a modeling and analysis of the crack growth behavior of rock during compression based on the colony growth hypothesis.

In this section, a general approach for modelling crack growth behavior under compression tests is provided. Firstly, the growth rate and the transformation rate of cracks are solved by introducing the crack growth model. Secondly, the growth direction of cracks is defined by the colony growth assumptions. Finally, the length increment of cracks is derived from the elastoplastic energy theory.

### The growth rate of cracks

In this paper, we explore the crack extension pattern under triaxial compression in experimental scale, thus a standard $100\,{\text{mm}} \times 50\,{\text{mm}}$ cylindrical rock sample is considered to build the crack growth model.

The main objects of study are the intrinsic microcracks inside porous rocks, that can randomly initiate, propagate and coalesce under a certain set of rules. In our research, the volumetric ratio of these fractured voxels is denoted as $C$, while $I$ is defined as the volumetric ratio of the non-fractured part (or rock skeletons). In this paper, volumetric ratio is the ratio of fracture part (cracks) or the non-fractured part (intact rock skeletons) to the overall rock volume. Thus, we propose a crack growth model based on the classical colony growth dynamics model^39^ as follows:

1 $$\left\{ {\begin{array}{*{20}l} {\frac{dC(t)}{{dt}} = \lambda I(t)C(t)} \hfill \\ {\frac{dI(t)}{{dt}} = - \lambda I(t)C(t)} \hfill \\ {I(t) + C(t) = 1} \hfill \\ \end{array} } \right.,$$

where $\lambda$ is the transformation rate between the volumetric ratio of fractured voxels and the volumetric ratio of rock skeletons.

Equation is a system of nonlinear ordinary differential equations, and the solution of the crack ratio $C(t)$ can be obtained by the separated variables method.

2 $$C(t) = \frac{{C_{0} e^{\lambda t} }}{{1 + C_{0} \left( {e^{\lambda t} - 1} \right)}},$$

where $C_{0}$ is the initial porosity and $C(t)$ is a logistic function which is commonly used to describe colony growth or changes in the number of species^40^. Analogically, the crack growth model aims to describe the changes in the amount of crack ratio. However, Eq. (2) is a monotonic increasing function, thus, this function can only simulate crack increase pattern after the crack closure stage where crack voxels ratio may drop a bit. Based on the crack growth model proposed in this paper, we mainly focus on the crack extension behavior after the crack closure stage.

By differentiating equation, we can obtain the growth rate of the fractured voxels as Eq. (3) which is used to quantify the proportion of intrinsic cracks over time.

3 $$C^{\prime}(t) = \frac{{\lambda C_{0} \left( {1 - C_{0} } \right)e^{\lambda t} }}{{\left( {1 - C_{0} + C_{0} e^{\lambda t} } \right)^{2} }}.$$

The prime in Eq. (3) means the derivative with respect to $t$. It is known that the crack increase rate will approach to the maximum at peak stress state in the triaxial compression state^36–38^. Therefore, by differentiating Eq. (3), we can obtain the followings:

4 $$C^{\prime\prime}(t) = \frac{{2C_{0}^{2} \lambda^{2} e^{2\lambda t} \left( {C_{0} - 1} \right)}}{{\left( {C_{0} e^{\lambda t} - C_{0} + 1} \right)^{3} }} - \frac{{C_{0} \lambda^{2} e^{\lambda t} \left( {C_{0} - 1} \right)}}{{\left( {C_{0} e^{\lambda t} - C_{0} + 1} \right)^{2} }}.$$

By solving $C^{\prime\prime}(t) = 0$, the crack growth rate $C^{\prime}(t)$ reaches the maximum at

5 $$t_{m} = \lambda^{ - 1} ln\left( {C_{0}^{ - 1} - 1} \right).$$

In this crack growth model, the peak stress moment $t_{m}$ is related to $C_{0}$ and $\lambda$, $C_{0}$ can be obtained by calculating the initial ratio of crack volume to the rock volume, but $\lambda$ is an hidden variable associated with several physical coefficients and the stress states of the rock. To calibrate the value of $\lambda$, the Coulomb Criterion is introduced to calculate the failure state of the rock samples. The Coulomb Criterion^41^ is shown as follows:

6 $$\left\{ {\begin{array}{*{20}l} {\left| \tau \right| = c + \sigma tan\phi } \hfill \\ {\sigma = \frac{1}{2}\left( {\sigma_{1} + \sigma_{2} } \right) + \frac{1}{2}\left( {\sigma_{1} - \sigma_{2} } \right)cos2\theta } \hfill \\ {\tau = \frac{1}{2}\left( {\sigma_{1} - \sigma_{2} } \right)sin2\theta } \hfill \\ \end{array} } \right.,$$

where $\sigma_{1}$ and $\sigma_{2}$ are the vertical and lateral (confining) stress, $c$ is the cohesion stress, $\phi$ is the internal friction angle of the rocks, $\theta = \pi /4 + \phi /2, \, \left( {\theta \in \left[ {0,\pi /2} \right]} \right)$ is the rupture angle, $\tau$ and $\sigma$ are the shear stress and normal stress.

Let the friction coefficient $f{\text{ = tan}}\phi$, the following equation can be derived from Eq. (6):

7 $$\left\{ {\left| \tau \right|{ - }f\sigma } \right\}_{max} = \frac{1}{2}\left( {\sigma_{1} - \sigma_{2} } \right)\sqrt {f^{2} + 1} - \frac{1}{2}\left( {\sigma_{1} + \sigma_{2} } \right)f = c.$$

Equation (7) is satisfied when the rock sample reaches its peak stress, therefore, according to the triaxial compressive test, $\sigma_{2}$ is a constant while the vertical stress is

8 $$\sigma_{1} = d\sigma_{1} t + \sigma_{2} ,$$

where $\sigma_{1}$ is the vertical stress with preloaded confining stress $\sigma_{2}$, $d\sigma_{1}$ is the loading rate which ranges from $0.5\sim 1.0Mpa/s$ and $t$ is the loading time.

By substituting Eq. (8) into Eq. (7), we can get:

9 $$\frac{1}{2}d\sigma_{1} t\sqrt {f^{2} + 1} - \frac{1}{2}\left( {d\sigma_{1} t + 2\sigma_{2} } \right)f = c,$$

then the moment of peak stress is obtained as:

10 $$t_{m} = \frac{{c + \sigma_{2} f}}{{\frac{1}{2}d\sigma_{1} \left( {\sqrt {f^{2} + 1} - f} \right)}},$$

Equation (10) is the peak stress moment calculated by Mohr Coulomb Criterion. Equation is the peak stress moment derived from our crack growth model. Thus, by making Eq. (10) equals to Eq. (5), the transformation rate $\lambda$ can be finally obtained as:

11 $$\lambda = \frac{{\frac{1}{2}d\sigma_{1} \left( {\sqrt {f^{2} + 1} - f} \right) \cdot ln(C_{0}^{ - 1} - 1)}}{{c + \sigma_{2} f}}.$$

**Figure 1:**
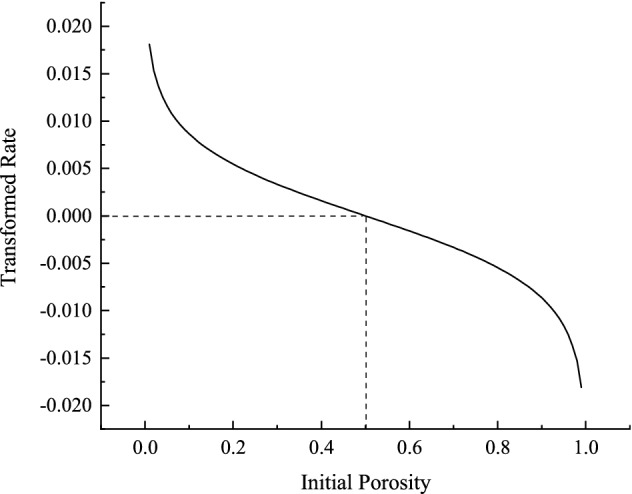
Transformation rate over initial porosity (in the case that $d\sigma_{1}$ = 0.5 Mpa/s, $\phi$ = 49.96$\circ$, $c$ = 17.19 Mpa and $\sigma_{2}$ = 5 Mpa).

The transformation rate $\lambda$ denotes the rate of conversion of a certain amount of rock skeletons to the fractured voxels, which is associated with the loading rate $d\sigma_{1}$, confining stress $\sigma_{2}$, cohesion $c$, friction angle $\theta$ and initial porosity $C_{0}$. Specifically, $\lambda$ is negatively proportional to the confining stress $\sigma_{2}$ and cohesion $c$. In other words, larger confining stress will lead to slower crack propagation, which accords with the laws of Eq. (10), indicating that larger confining stress prolongs the time to reach the peak stress. Similarly, the cohesion $c$ of the rock reveals the same pattern. Furthermore, increasing the loading rate or decreasing the initial porosity will promote the growth of cracks. It is worth noting that the initial porosity should not be greater than 0.5, otherwise the transformation rate will be negative (see Fig. 1). The effect of friction angle $\phi$ ($f{\text{ = tan}}\phi$) on the transformation rate $\lambda$ is not obvious, but we can rewrite the right part of Eq. (11) as:

12 $$\lambda \propto \frac{1}{{\left( {Af + B} \right) \cdot \left( {\sqrt {f^{2} + 1} + f} \right)}},$$

where $A$ and $B$ are positive constants when the initial porosity $C_{0}$ is less than 0.5 (the initial porosity is less than 0.5 in most cases for porous rock samples). Therefore, the transformation rate $\lambda$ is an inverse proportional function of the friction angle $\phi$ ($f{\text{ = tan}}\phi$), namely, larger $\phi$ will lead to slower extension rate of fractured voxels.

### The growth direction of cracks

In this paper, the evolution of fracture voxels is considered as a natural colony growth behavior. Therefore, the growth direction (behavior) of the crack is assumed to be controlled by its own properties and the surrounding environment. To be specific, the growth directions of crack tips are subjected to the following assumptions:

1. This paper assumes that the crack propagation direction is influenced by its own length, that is, longer cracks may tend to expand at both tips along their original heading direction.
2. The growth direction of each crack is also affected by the surrounding cracks, namely, the current crack tip may be attracted by other adjacent crack tips and tend to coalesce with other crack tips.
3. The growth direction is also controlled by the Mohr Coulomb Criterion, thus, this paper assumes that the rupture angle $\theta$ defined in Mohr Coulomb Criterion also affects the propagation path of the crack tip.
4. This paper assumes that the growth direction of the crack tip is attracted to align with the stress path generated by the vertical stresses acting on the top and bottom of the rock specimen.

**Figure 2:**
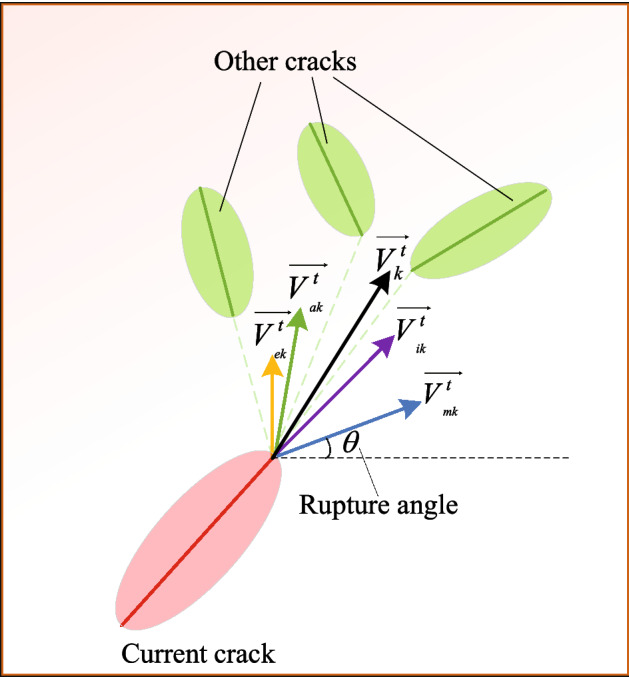
2D schematic diagram of the growth direction of cracks ($\overrightarrow {{V_{{_{ik} }}^{t} }}$ is the inertial direction, $\overrightarrow {{V_{{_{ak} }}^{t} }}$ is the attractive direction, $\overrightarrow {{V_{{_{mk} }}^{t} }}$ is the Coulomb direction, $\overrightarrow {{V_{{_{ek} }}^{t} }}$ is the edge attraction direction, $\overrightarrow {{V_{k}^{t} }}$ is the resultant direction).

Therefore, the growth direction in this paper is assumed to be defined as the followings (see Fig. 2):

13 $$\overrightarrow {{V_{k}^{t} }} = \alpha_{{_{1k} }}^{t} \overrightarrow {{V_{{_{ik} }}^{t} }} + \alpha_{{_{2k} }}^{t} \overrightarrow {{V_{{_{ak} }}^{t} }} + \alpha_{{_{3k} }}^{t} \overrightarrow {{V_{{_{mk} }}^{t} }} { + }\alpha_{{_{4k} }}^{t} \overrightarrow {{V_{{_{ek} }}^{t} }} ,$$

where $\overrightarrow {{V_{k}^{t} }}$ is the growth direction of the $kth$ crack tip at time $t$, $k \in [1,N]$, $N$ is the total number of cracks, $t \in [0,T]$.

In Eq. (13), the first component $\overrightarrow {{V_{{_{ik} }}^{t} }}$ represents the direction of inertia, which is affected by the former state of the $kth$ crack at time $t - 1$. That is, the $kth$ crack tends to propagate in its original direction due to its inertia. $\overrightarrow {{V_{{_{ik} }}^{t} }}$ can be expressed as the difference in coordinates between one time slot.

14 $$\overrightarrow {{V_{{_{ik} }}^{t} }} = \frac{{\left( {x_{k}^{{t{ - }1}} { - }x_{k}^{t} ,y_{k}^{{t{ - }1}} { - }y_{k}^{t} ,z_{k}^{{t{ - }1}} { - }z_{k}^{t} } \right)}}{{\left\| {x_{k}^{{t{ - }1}} { - }x_{k}^{t} ,y_{k}^{{t{ - }1}} { - }y_{k}^{t} ,z_{k}^{{t{ - }1}} { - }z_{k}^{t} } \right\|}},$$

where $\alpha_{{_{1k} }}^{t}$ in Eq. (13) is the inertial weight, denoted as:

15 $$\alpha_{{_{1k} }}^{t} = \frac{{l_{{_{k} }}^{t} }}{{\mathop {max}\limits_{k \in [1,N]} \left( {l_{{_{k} }}^{t} } \right)}},$$

where $l_{{_{k} }}^{t}$ is the length of the $kth$ crack at time $t$. From Eq. (15), the inertial weight is positively proportional to the length of the crack, that is, macroscale cracks tend to develop in their own inertial direction and are less likely to be influenced by their surrounds (i.e. other cracks). It is also evident from the experimental tests that the failure of the rock is caused by a small fraction of macroscale cracks that can dominate their own directions to the greatest possible extent without affecting by other small-scale cracks.

The second part of Eq. (13) $\overrightarrow {{V_{{_{ak} }}^{t} }}$ denotes the attractive direction, which indicates that within the neighborhood $\mathop U\limits^{ \circ } (p_{k}^{t} ,\delta ) = \left\{ {\left. {\mathop {p_{j}^{t} }\limits_{j \in [1,N],j \ne k} } \right|0 < d(p_{j}^{t} ,p_{k}^{t} ) < \delta } \right\}$ of the current crack tip $p_{k}^{t}$, all crack tips in the neighborhood will attract the current crack tips $p_{k}^{t}$ towards the resultant “force” generated. This component reveals that the crack growth model treats each crack as an individual object which can be affected by others. The form of $\overrightarrow {{V_{{_{ak} }}^{t} }}$ can be written as:

16 $$\overrightarrow {{V_{{_{ak} }}^{t} }} { = }\frac{{\left( {\sum\limits_{j \in [1,N],j \ne k} {\omega_{{_{j} }}^{t} (x_{k}^{t} - x_{{_{j} }}^{t} ),} \sum\limits_{j \in [1,N],j \ne k} {\omega_{{_{j} }}^{t} (y_{k}^{t} - y_{{_{j} }}^{t} ),} \sum\limits_{j \in [1,N],j \ne k} {\omega_{{_{j} }}^{t} (z_{k}^{t} - z_{{_{j} }}^{t} )} } \right)}}{{\left\| {\sum\limits_{j \in [1,N],j \ne k} {\omega_{{_{j} }}^{t} (x_{k}^{t} - x_{{_{j} }}^{t} ),} \sum\limits_{j \in [1,N],j \ne k} {\omega_{{_{j} }}^{t} (y_{k}^{t} - y_{{_{j} }}^{t} ),} \sum\limits_{j \in [1,N],j \ne k} {\omega_{{_{j} }}^{t} (z_{k}^{t} - z_{{_{j} }}^{t} )} } \right\|}},$$

where $\omega_{j}^{t} = e^{{ - p\left( {s_{j}^{k} } \right)^{2} }}$ represents the attractive strength of each crack tip $p_{j}^{t}$ within the neighborhood $\mathop U\limits^{ \circ } (p_{k}^{t} ,\delta )$, $s_{j}^{k} \in (0,\delta ]$ is the attractive radius showing the distance between the current crack tip $p_{k}^{t}$ and one of the neighboring crack tip $p_{j}^{t}$, $p$ is the attenuation factor which ranges from 0 to 1. It is noticeable that the attracting strength $\omega_{j}^{t}$ is a Weibull-form function whose value is negatively proportional to the attractive radius $s_{j}^{k}$.

$\alpha_{{_{2k} }}^{t}$ in Eq. (13) is the attractive weight written as:

17 $$\alpha_{{_{2k} }}^{t} = 1 - \frac{1}{{1 + u_{{_{k} }}^{t} }},$$

where $u_{{_{k} }}^{t} = \left\| {\sum\nolimits_{j \in [1,N],j \ne k} {\left( {x_{k}^{t} - x_{{_{j} }}^{t} } \right),} \sum\nolimits_{j \in [1,N],j \ne k} {\left( {y_{k}^{t} - y_{{_{j} }}^{t} } \right),} \sum\nolimits_{j \in [1,N],j \ne k} {\left( {z_{k}^{t} - z_{{_{j} }}^{t} } \right)} } \right\|$ which represents the magnitude of the sum of vectors of the current crack tips $p_{k}^{t}$ pointing to all other crack tips $p_{j}^{t}$ within the neighborhood $\mathop U\limits^{ \circ } (p_{k}^{t} ,\delta )$. Thus, larger $u_{{_{k} }}^{t}$ will lead to a larger $\alpha_{{_{2k} }}^{t}$, implying that the current crack tip tends to expand toward the region with more cracks.

The third part $\overrightarrow {{V_{{_{mk} }}^{t} }}$ in Eq. (13) is the Coulomb direction, that is, the crack extension direction is also controlled by the Coulomb Criterion, which drives the crack in the direction of the rupture angle $\theta$. The Coulomb Criterion states that in triaxial compression tests, once the shear stress is greater than the shear strength of the specimen, the specimen is susceptible to damage and the fracture pattern of the crack is mostly consistent with the rupture angle. In this regard, it is assumed in this paper that the fracture behavior of each crack on the microscopic level also conforms to the Coulomb criterion to some extent. Therefore, $\overrightarrow {{V_{{_{mk} }}^{t} }}$, as another control component of growth direction, can be written in the following form:

18 $$\overrightarrow {{V_{mk}^{t} }} = \frac{{\overrightarrow {{V_{ik}^{t} }} \cdot cos\left| {\left( {\frac{\pi }{2} - \theta } \right) - \xi_{{_{k} }}^{t} } \right|}}{{\left\| {\overrightarrow {{V_{ik}^{t} }} \cdot cos\left| {\left( {\frac{\pi }{2} - \theta } \right) - \xi_{{_{k} }}^{t} } \right|} \right\|}},$$

Equation (18) indicates that the Coulomb direction is derived from the inertial direction by rotating an angle $\xi_{{_{k} }}^{t}$ that is between the inertial direction of the crack tip and the vertical stress.

$\alpha_{{_{3k} }}^{t}$ is the Coulomb weight defined as:

19 $$\alpha_{{_{3k} }}^{t} { = }cos\left| {\left( {\frac{\pi }{2} - \theta } \right) - \xi_{{_{k} }}^{t} } \right|.$$

$\alpha_{{_{3k} }}^{t}$ is a decreasing function in the interval of $\left[ {0,\frac{\pi }{2}} \right]$, which indicates that the crack is more likely to be affected by the Coulomb Criterion when its direction is approaching to the rupture angle.

In Eq. (13), the last part $\overrightarrow {{V_{{_{ek} }}^{t} }}$ is the edge attraction direction, which can be interpreted as the vertical stress acting on the top and bottom edge of the specimen will create a stress path perpendicular to the edge and the cracks will be attracted to aligned with the stress path. The edge attraction direction is defined as:

20 $$\overrightarrow {{V_{{_{ek} }}^{t} }} = \left\{ {\begin{array}{*{20}c} {(0,0,1), \, if \, D_{ku}^{t} \le D_{kb}^{t} } \\ {(0,0, - 1), \, if \, D_{ku}^{t} > D_{kb}^{t} } \\ \end{array} } \right.,$$

where $D_{ku}^{t}$ and $D_{kb}^{t}$ are the distance of the $kth$ crack tip to the up and the bottom edge of the specimen respectively at time $t$. Equation (20) indicates that the crack tip is likely to propagate towards the nearest edge.

$\alpha_{{_{4k} }}^{t}$ represents the edge weight written as:

21 $$\alpha_{{_{4k} }}^{t} = \frac{{H/2 - min(D_{ku}^{t} ,D_{kb}^{t} )}}{H/2}.$$

Equation (21) manifests that the edge attraction direction is more likely to dominate the crack growth direction when the crack is close to the edge.

In summary, different components of Eq. (13) disclose different influences on the fracture behavior of each crack. The first component reflects the influence of the current state; the second component reveals the influence of the surrounding cracks; the third component implies the constitutive law of fracture behavior, and the last part indicates the surrounding stress environment generated by the external forces. Therefore, the overall fracture behavior of each crack is controlled by equation defined by the aforementioned assumptions.

### The definition of energy and increment of cracks

Crack expansion in rocks can be explained from the perspective of energy theory, i.e. the energy absorbed by the crack tip exceeds its own limit causing the crack to start expanding outward. The energy absorbed by the rock sample at time $t$ is defined as $E_{all}^{t}$, Since rock samples behave differently under different compression stages such as crack closure stage, elastic stage, plastic stage and failure stage. In this paper our model considers only two circumstances of compression behavior, that is the linear condition without fracture occurred and the nonlinear condition with fracture event. The model merges the crack closure stage into the elastic stage by ignoring the nonlinear behavior of cracks. The reason for that is because we mainly focus on the fracturing behavior of hard dense rocks such as granite which contains very few defects and thus leading to very insignificant crack closure manifestation. In other point, crack closure behavior is quite complex by introducing into the differential dynamical equations. The analytical solution for crack growth in this stage cannot be found. Therefore, we simplify the compression process into two main stages in our model.

On the basis of elastic mechanics theory, the absorbed energy in the linear elastic stage can be expressed as:

22 $$E_{all}^{t} \, = \,\frac{1}{2} \cdot \left( {\sigma_{{_{x} }}^{t} \cdot \varepsilon_{{_{x} }}^{t} + \sigma_{{_{y} }}^{t} \cdot \varepsilon_{{_{y} }}^{t} + \sigma_{{_{z} }}^{t} \cdot \varepsilon_{{_{z} }}^{t} } \right),$$

where

23 $$\left\{ \begin{gathered} \varepsilon_{x}^{t} = \frac{1}{E}\left( {\sigma_{x}^{t} - \nu \left( {\sigma_{y}^{t} + \sigma_{z}^{t} } \right)} \right) \hfill \\ \varepsilon_{y}^{t} = \frac{1}{E}\left( {\sigma_{y}^{t} - \nu \left( {\sigma_{x}^{t} + \sigma_{z}^{t} } \right)} \right) \hfill \\ \varepsilon_{z}^{t} = \frac{1}{E}\left( {\sigma_{z}^{t} - \nu \left( {\sigma_{y}^{t} + \sigma_{x}^{t} } \right)} \right) \hfill \\ \end{gathered} \right.,$$

$\varepsilon_{{_{x} }}^{t}$, $\varepsilon_{{_{y} }}^{t}$ and $\varepsilon_{{_{z} }}^{t}$ denote the triaxial elastic strain at time $t$, $E$ is the elastic modules of the rock sample.

Plastic strain energy and fracture energy should be taken into account in the nonlinear phase since this is when the cracks begin to propagate. The absorbed energy $E_{all}^{t}$ in this phase may be written as.

24 $$E_{all}^{t} \, = \,E_{e}^{t} + E_{p}^{t} + E_{f}^{t} ,$$

where

25 $$\left\{ \begin{gathered} E_{e}^{t} = \frac{1}{2} \cdot \left( {\sigma_{{_{x} }}^{t} \cdot \varepsilon_{{_{x} }}^{t} + \sigma_{{_{y} }}^{t} \cdot \varepsilon_{{_{y} }}^{t} + \sigma_{{_{z} }}^{t} \cdot \varepsilon_{{_{z} }}^{t} } \right) \hfill \\ E_{p}^{t} = \frac{1}{2} \cdot \left( {\sigma_{{_{x} }}^{t} \cdot \varepsilon_{{_{px} }}^{t} + \sigma_{{_{y} }}^{t} \cdot \varepsilon_{{_{py} }}^{t} + \sigma_{{_{z} }}^{t} \cdot \varepsilon_{{_{pz} }}^{t} } \right) \hfill \\ E_{f}^{t} = \sum\limits_{k \in [1,N]} {\left( {\sigma_{{_{k} }}^{{t{ - }1}} - \mu c} \right) \cdot \delta {\mathcal{V}}_{k}^{{t{ - }1}} } \hfill \\ \end{gathered} \right..$$

In Eq. (25), $E_{e}^{t}$, $E_{p}^{t}$ and $E_{f}^{t}$ represent the elastic energy, the plastic energy and fracture energy respectively. $\varepsilon_{px}^{t}$, $\varepsilon_{py}^{t}$ and $\varepsilon_{pz}^{t}$ are the triaxial plastic strain at time $t$, which can be defined as:

26 $$\left\{ \begin{gathered} \varepsilon_{pz}^{t} = \frac{{\delta {\mathcal{V}}^{t - 1} }}{{4\pi R^{2} H}} \hfill \\ \varepsilon_{py}^{t} = \frac{{\delta {\mathcal{V}}^{t - 1} }}{{4\pi R^{3} tan\theta }} \hfill \\ \varepsilon_{px}^{t} = \frac{{\delta {\mathcal{V}}^{t - 1} }}{{4\pi R^{3} tan\theta }} \hfill \\ \end{gathered} \right.,$$

where $\delta {\mathcal{V}}^{{t{ - }1}}$ is the total volume increment of the cracks at time $t - 1$ (see Eq. (27)), $H$ and $R$ are the height and radius of the rock sample.

27 $$\delta {\mathcal{V}}^{{t{ - }1}} { = }V(C - C_{0} ),$$

where $V$ is the volume of the rock specimen.

**Figure 3:**
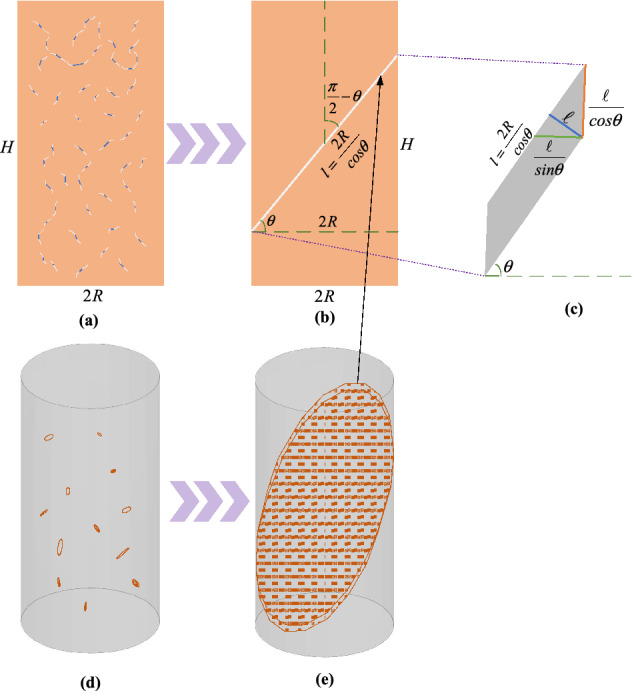
The transformation of the crack incremental voxel into an equivalent elliptical thin plane. (**a**) is a two-dimensional schematic of the fractured voxel; (**b**) is a front view of the transformed elliptical thin plane; (**c**) is a schematic enlargement of the elliptical thin plane; (**d**) is a three-dimensional schematic of the fractured voxel, and (**e**) is a three-dimensional schematic of the transformed elliptical thin plane.

Equation (26) assumes that the volume increment of cracks will be transformed into the plastic deformation of the rock sample, therefore, for simplicity and idealization, our model converts the volume increment of cracks into the equivalent volume of a thin plane with an inclination of $\theta$ defined by Mohr Coulomb Criterion and a thickness of $\ell$, as illustrated in Fig. 3.

In Fig. 3a, the blue segments are the initial microcracks while the white segments are the crack increment that can be converted into an equivalent thin plane with a total volume of $\delta {\mathcal{V}}$ as shown in Fig. 3b. The length of equivalent plane can be derived as:

28 $$l = \frac{2R}{{cos\theta }},$$

according to the schematic of equivalent plane in Fig. 3c, the equivalent plane is an elliptical thin sheet, thus, the thickness of the plane can be obtained as:

29 $$\ell = \frac{{\delta {\mathcal{V}}}}{2\pi Rl}.$$

Therefore, the equivalent vertical and lateral increment caused by the crack increment can be written as:

30 $$\left\{ {\begin{array}{*{20}c} {\delta v = \frac{\ell }{cos\theta }} \\ {\delta h = \frac{\ell }{sin\theta }} \\ \end{array} } \right..$$

By combining equation (28)–(30), the vertical and lateral plastic strain of the rock sample can be derived in the form of Eq. (31) which is equivalent to equation.

31 $$\left\{ \begin{gathered} \varepsilon_{pv} = \frac{{\delta {\mathcal{V}}}}{{4\pi R^{2} H}} \hfill \\ \varepsilon_{ph} = \frac{{\delta {\mathcal{V}}}}{{4\pi R^{3} tan\theta }} \hfill \\ \end{gathered} \right..$$

In Eq. (25), $\delta {\mathcal{V}}_{k}^{{t{ - }1}}$ is the increment volume of the $kth$ crack, $\mu$ is the coefficient of friction which is defined in Ref.^1^ and $\sigma_{{_{k} }}^{{t{ - }1}}$ is defined as the initial fracture stress whose orientation is consistent with the shear stress on each crack. Inspired by Ref.^1^, $\sigma_{k}^{{t{ - }1}}$ can be expressed in Eq. (32).

32 $$\left\{ {\begin{array}{*{20}c} {\sigma_{{_{k} }}^{{t{ - }1}} { = }\left( {\tau_{{_{kr} }}^{t - 1} - \mu p_{{_{k} }}^{t - 1} } \right)} \\ {\tau_{{_{kr} }}^{t - 1} = \frac{1}{2}\left( {\sigma_{1}^{{t{ - }1}} - \sigma_{2} } \right)sin2\theta_{{_{k} }}^{t - 1} } \\ {p_{{_{k} }}^{t - 1} = \frac{1}{2}\left[ {\left( {\sigma_{1}^{{t{ - }1}} + \sigma_{2} } \right) + \left( {\sigma_{1}^{{t{ - }1}} - \sigma_{2} } \right)cos2\theta_{{_{k} }}^{t - 1} } \right]} \\ \end{array} } \right..$$

**Figure 4:**
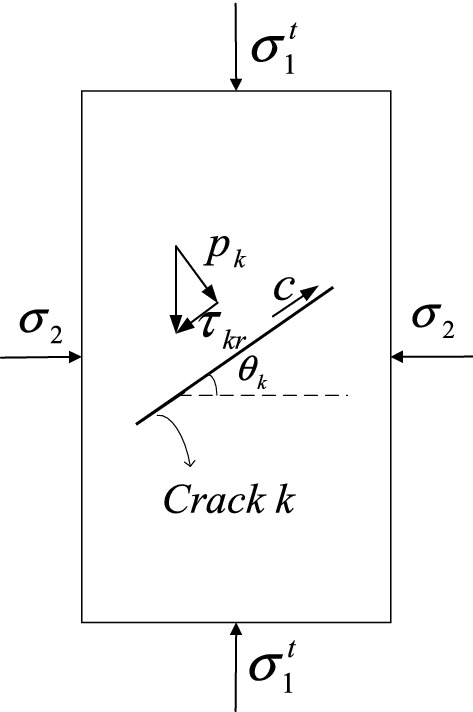
Schematic diagram of a particular crack $i$ inside the rock sample subjected to compression.

As indicated in Fig. 4, $\tau_{kr}$ and $p_{k}$ are the resolved shear stress and the normal stress on the face of crack $k$, and the direction of the crack $k$ with respect to the horizontal axis is defined as $\theta_{k}$. $\sigma_{{_{1} }}^{{t{ - }1}}$ is axial stress and $\sigma_{2}$ is the confining stress. In this paper, the fracture energy is defined as the work done by the crack tip to extend over a certain distance by overcoming the cohesion. It is noticeable that as the increase of the confining stress $\sigma_{2}$, the initial fracture stress $\sigma_{k}$ will need more time to surpass cohesion and finally initiate the fracture behavior. This is aligned with Eq. (13) that higher confining stress will cause result in a lower conversion rate of rock skeletons to the fractured voxels. It is noticeable that, the failure nature of rock samples here is purely shearing failure because the direction of cohesion in Fig. 4 is parallel to the fracture surface.

In Eq. (25), the overall fracture energy of rock specimen gained from the triaxial compression is obtained, and accordingly, the fracture energy of the $kth$ crack at time $t$ can be evaluated based on the length of its longest axis, which can be defined as:

33 $$E_{k}^{t} = E_{f}^{t} \cdot \frac{{l_{k}^{t} }}{{\sum\nolimits_{k \in [1,N]} {l_{k}^{t} } }}.$$

where $l_{k}^{t}$ is the length of $kth$ crack at time $t$.

At this point, it is inevitable to know the unit energy required for each crack to achieve unit length growth. Therefore, this paper introduced the unit resistant energy of each crack as $R_{k}^{t}$:

34 $$R_{k}^{t} = crw,$$

where, $r$ and $w$ are the thickness and width of cracks respectively.

Therefore, the crack increment at time $t{ + }1$ can be written as:

35 $$\delta l_{k}^{{t{ + }1}} = \frac{{E_{k}^{t} }}{{R_{k}^{t} }},$$

## Numerical simulation and analysis

**Figure 5:**
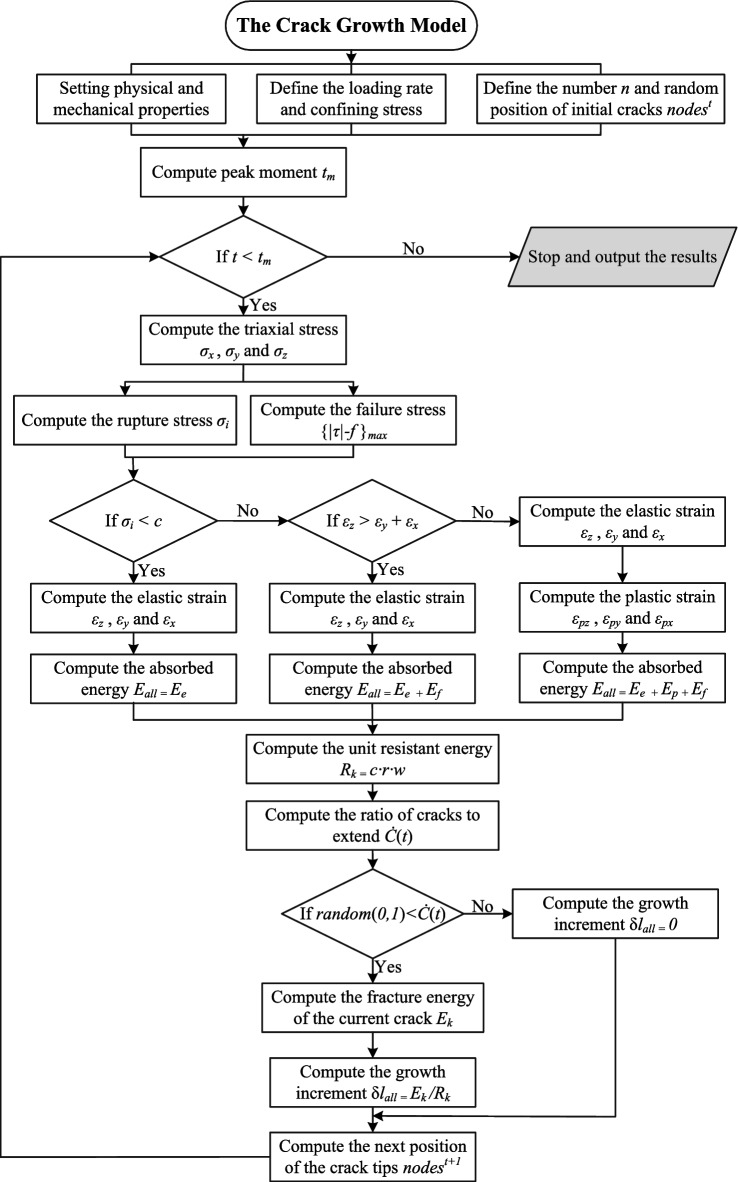
Simulation flowchart of the crack growth model.

Based on the crack growth model presented in “Methodology”, the numerical simulation flow is presented in Fig. 5. In this section, we first introduce the basic configuration of the numerical simulation using MATLAB. Secondly, our simulation results are validated against experimental results and PFC3D results to demonstrate the effectiveness of our method. In addition, this section provides a detailed analysis of the simulation results and key parameters.

### Model configuration

The proposed model elaborated in “Methodology” is neither a FEM model nor DEM model, which cannot be simulated by any commercial academic software. Thus, our model is built and verified with MATLAB 2020a.

**Figure 6:**
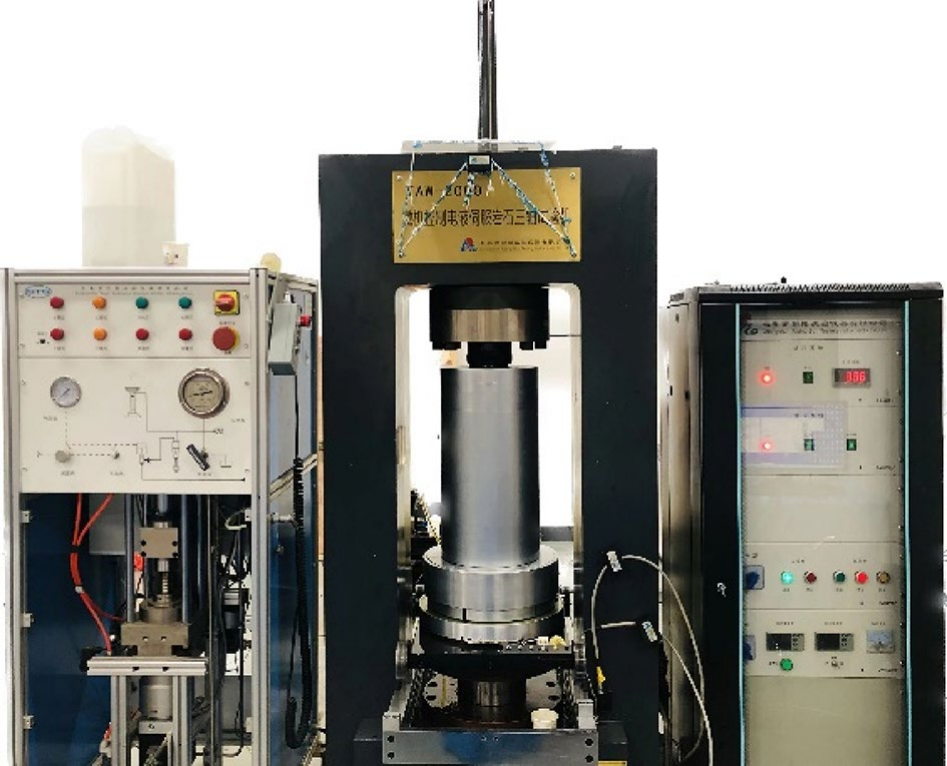
Test equipment TAW2000.

This paper simulates the fracture behavior of standard cylindrical granites with a size of $100\,{\text{mm}} \times 50\,{\text{mm}}$ in Sanshandao under compression without or with confining stress. The physical and mechanical properties of the granites are obtained from real granite samples in Sanshandao golden mine, The main mineral composition of this granite is plagioclase, potassium feldspar, quartz and black mica, with 44%, 20%, 32% and 4% volume fractions, respectively. The grain size of the main minerals ranged from 2 to 4 mm. The rock samples were processed into cylindrical specimens of $\phi$ 50 mm × 100 mm, which were subjected to uniaxial and triaxial compression tests. The uniaxial compression tests and the triaxial compression test were carried out using a TAW2000 microcomputer control electrohydraulic servo rock triaxial testing machine (Fig. 6) at Hebei University of Engineering, with a loading rate of 0.5 MPa/s. The test was controlled by a circumferential extensometer. The mechanical properties obtained are shown in Table 1.

**Table. 1.** Physical and mechanical properties of Sanshandao granites.

Density (kg m^−3^)	Compressive elastic modulus (GPa)	Possion’s ratio	Friction angle ($^\circ$)	Cohesion (MPa)
2686	43.98	0.20	49.96	17.19

Therefore, in this study, the crack evolution behavior of standard cylindrical granite samples was simulated with confining stresses of 0, 5, 10, 20 and 30 Mpa respectively. The axial load is applied at a loading rate of 0.5 Mpa/s. A number of initial intrinsic microcracks ranging from 800 to 1000 with a thickness of 0.05 mm were randomly distributed within the rock samples, each with an initial length of 0.1–0.5 mm.

### Validation with experimental results and PFD3D results

**Figure 7:**
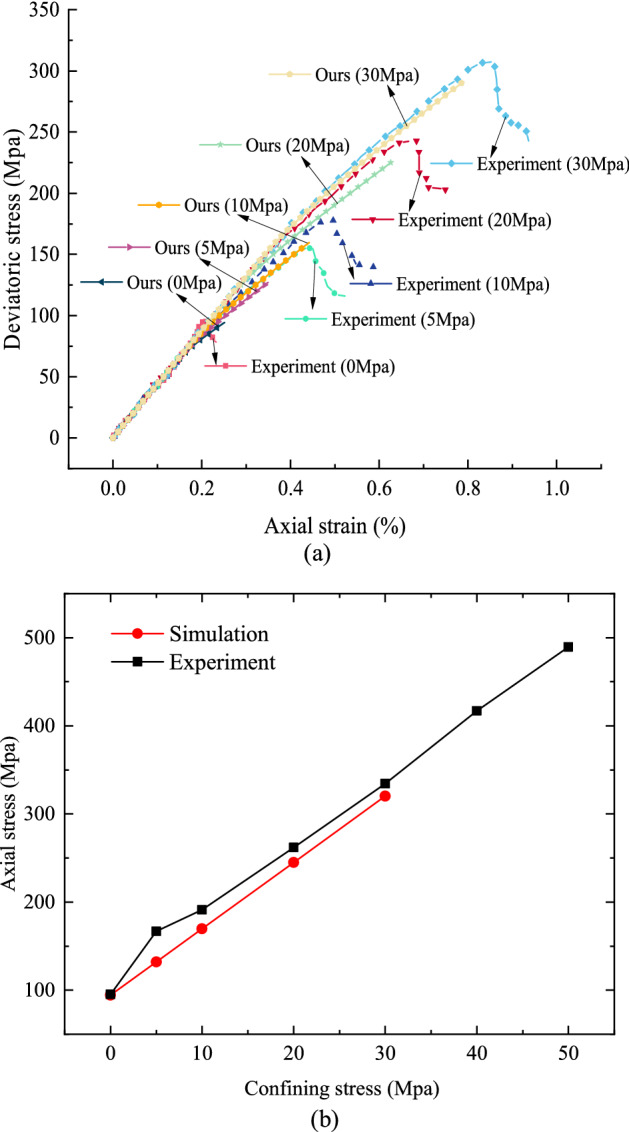
Compression test results of granite obtained from experiments and our simulation model. (**a**) Stress–strain curves of compression tests and simulation results; (**b**) Comparison of the strength envelops of the experimental results and simulation results under different confining stress.

Figure 7a elucidates the compression results of the experiment and our proposed model, which shows that the simulation results are close enough to the experimental results in terms of peak stress and elastic modulus. Figure 7b also demonstrates the strength envelops of the simulated and experimental results under different confining stresses, and it is noticeable that the simulated results agree well with the experimental results. Furthermore, by substituting Eq. (10) into Eq. (8), we can obtain the peak axial stress as:

36 $$\sigma_{m} = \left( {\sqrt {f^{2} + 1} + f} \right)^{2} \cdot \sigma_{2} + \left( {\sqrt {f^{2} + 1} + f} \right) \cdot 2c,$$

It can be seen that the peak axial strength $\sigma_{m}$ is positively proportional to the confining stress $\sigma_{2}$, which is also align with the plot in Fig. 7b. We can let

37 $$p = \left( {\sqrt {f^{2} + 1} + f} \right)^{2}$$

and

38 $$q = \left( {\sqrt {f^{2} + 1} + f} \right) \cdot 2c,$$

$p$ is slope of strength envelop over confining stresses and $q$ is the uniaxial strength. The slope $p$ is positively associated with the internal friction angle $\phi \in \left( {0,\frac{\pi }{2}} \right)$, and the value region for $p$ is $(1,\infty )$. $q$ is positively proportional to the internal friction angle $\phi$ and the cohesion $c$.

**Figure 8:**
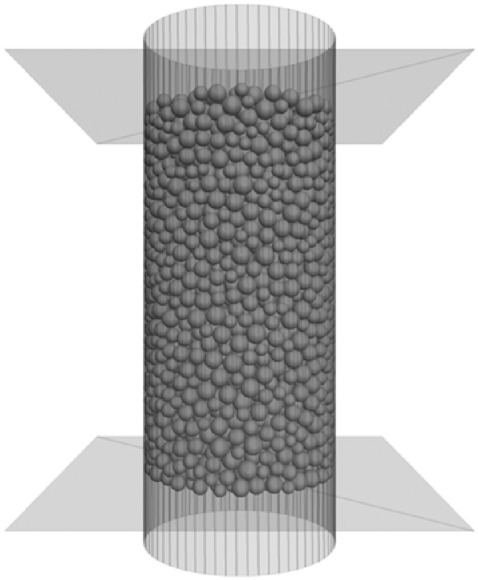
Particle model constructed with PFC3D.

To further validate the effectiveness of our method, a discrete-element PFC3D numerical model is built in this paper to perform triaxial compression tests. In modeling stage, the geometry and physical properties are set to be equal to the laboratory conditions. In this manner, the simulation result should be as close as possible to the laboratory test result to the possible extent after repeated trial tests. In the PFC simulation, the calibrated parameter setting is listed in Table 2. The PFC3D model is displayed in Fig. 8.

**Table 2:** Mechanical parameters of the PFC3D model.

Parameter	Symbol	Quantity
Contact modulus of the particle	$E/{\text{GPa}}$	122
Radius factor of the parallel bond	$\lambda$	1.0
Ratio of normal to stiffness of the parallel bond	$\overline{k}^{n} /\overline{k}^{s}$	1.33
Particle friction coefficient	$\mu$	0.5
Parallel-bond tensile strength, mean	$\sigma_{t} /{\text{Mpa}}$	226
Cohesion	$c/{\text{Mpa}}$	226
Minimum particle radius	$r_{\min } /{\text{mm}}$	1.5
Maximum particle radius	$r_{\max } /{\text{mm}}$	2.5
Internal friction angle	$\phi /^{ \circ }$	15
Density	$\rho /{\text{kg}}\,{\text{m}}^{ - 3}$	2686

**Figure 9:**
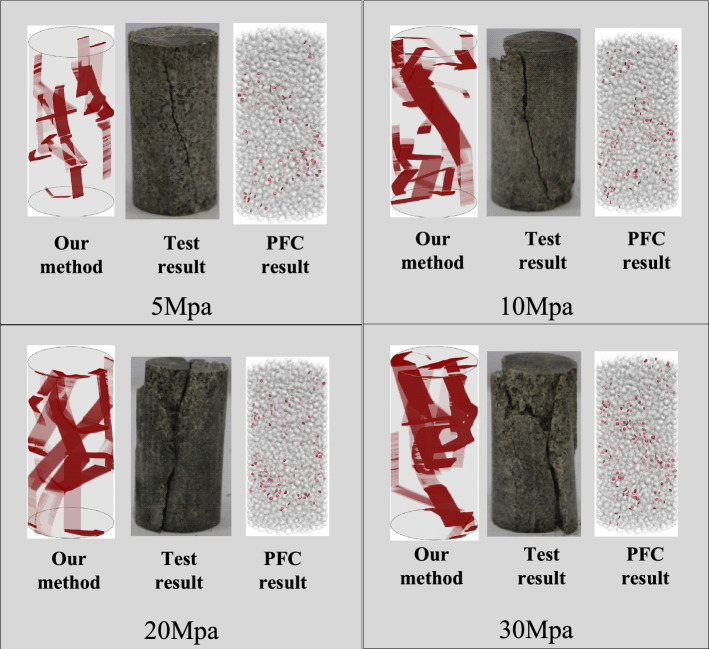
Comparison of our simulation result with the experimental test results and PFC3D results at peak stress state.

Figure 9 shows the simulated fracture evolution of the rock samples under different confining stresses. As depicted in Fig. 9, the results of laboratory tests and simulations of our method are also presented. The comparison results show that the crack morphology of the different methods agrees to some extent with the Coulomb criterion, i.e., microcracks propagate and coalesce mainly in the diagonal region, forming macroscopic cracks penetrating the specimens. However, despite the main crack path, our crack growth model can simulate many other tiny crack paths that are concentrated around the main crack path, which means that our model can exhibit a certain degree of randomness within the constraints of the Coulomb criterion. Furthermore, PFC3D model cannot well present the fracture pattern that the failure plane is relatively aligned with the axial stress at lower confining stresses.

### Simulation result and analysis

**Figure 10:**
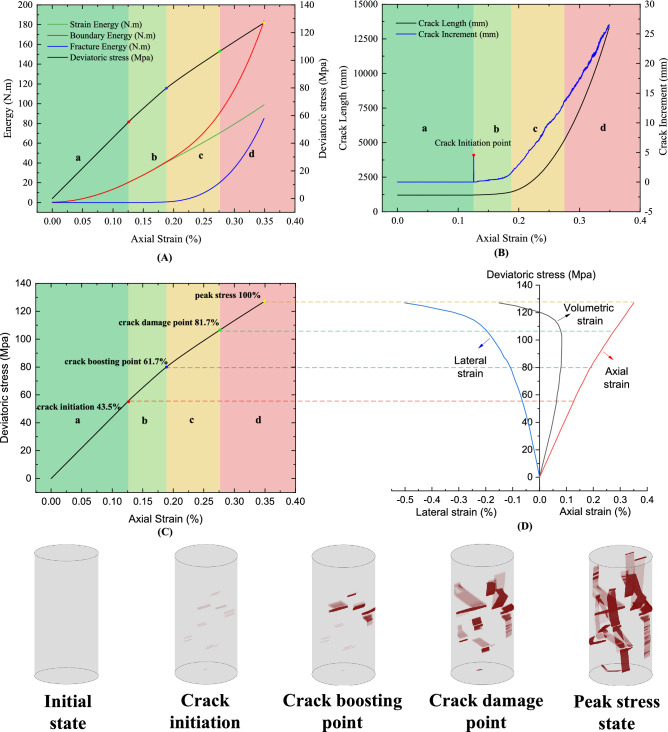
Simulated results with the proposed simulation method with 5 Mpa confining stress. (**A**). The deviatoric stress–strain curve and energy curves for which the boundary energy is the sum of the fracture energy and the strain energy; (**B**). The crack length and crack increment over axial strain; (**C**). The deviatoric stress–strain curve; (**D**). The deviatoric stress curves over axial, lateral and volumetric strain.

Figure 10 illustrates the simulated result under 5Mpa confining stress, which can be divided into four stages (a), (b), (c), and (d), i.e., elastic stage, crack initiation stage, stable growth stage, and unstable growth stage, according to the marked points in Fig. 10B,C. Figure 10A recorded the deviatoric stress/strain curve and the energies absorbed during the compression process. In this paper, strain energy, fracture energy and the boundary energy are monitored separately along with the stress/strain curve. The boundary energy here is refer to the sum of the strain energy and the fracture energy.

The simulation results of the triaxial compression test for other cases are quite similar to that shown in Fig. 10, and therefore, no additional elaboration will be involved. Meanwhile, the differences in different cases will be discussed in the later section.

Stage (a) is the elastic stage where the compression-induced fracture has not yet occurred, as can be seen in Fig. 10B, where the increment of fracture inside the rock sample remains zero. Additionally, Fig. 10A shows that the axial stress–strain curve increases rapidly and almost linearly due to the complete conversion of the accumulated energy in this phase into strain energy. Figure 10D also indicates that the rock sample is gradually and linearly compressed as the axial strain, lateral strain and the volumetric strain change linearly.

Stage (b) is the crack initiation stage, where the cracks initiate and develop at a relatively steady and slow rate. As can be seen in Fig. 10A,B, the fracture energy and crack length are increasing at a very insignificant speed. At this stage, the crack growth leads to a gradual change in lateral strain from a linear to a nonlinear variation, which counteracts the axial compressive strain and leads to a deceleration of the increase of volumetric strain toward compressive deformation. As can be seen in Fig. 10D, the reduction rate of volumetric strain is significantly slower after the crack initiation point in which the axial stress is at around 43.5% of the peak stress. It is noteworthy that at the beginning of this stage, the crack increment will surge sharply and fall back (see Fig. 10B). The crack increment is the increment of crack length per unit time, that is, it is the derivative of crack length growth. This phenomenon can be explained by the fact that once the axial stress exceeds the initial fracture stress of the crack, the initial crack will be initiated explosively by releasing the elastic strain energy stored in the elastic phase.

Stage (c) is the stable growth stage, in which the crack length and the fracture energy increase rapidly and stably, forcing the volumetric strain towards expansion rather than compaction. It can be seen from Fig. 10C that this stage starts from the crack boosting point at about 61.7% of the peak stress. At this stage, volumetric variation is not very significant either in compaction or expansion, while this stage witnesses a significant growth of cracks from the crack boosting point to the crack damage state as demonstrated in Fig. 10B.

Stage (d) is the unstable growth stage which starts from the crack damage point (around 81.7% of the peak stress) which is the transition point of the volumetric strain from compaction to expansion and ends at the peak stress. In this stage, the crack increment exhibits relatively strong jitter and the crack growth rate peaks, leading to a sharp increase in all indicators.

It can be seen that the numerical results are relatively reasonable and accord with experimental results in Refs.^36,37^ and the PFC simulation result in Ref.^38^. In particular, it is well accepted that the crack initiation point is at around 40% of the peak stress and the crack damage point is at around 80%^36,37^, which is very close to our simulation results.

Additionally, according to the simulation of the fracture behavior of the cylindrical sample in Fig. 10, it can be found that fracture has local effect, that is, the crack growth may first appear locally in a certain region and then propagate outward. Specifically, during stage (b), cracks initiate from the center of the cylindrical sample and propagate outwards to the bottom and upper position of the sample. In stage (c), most of the cracks extensively develop in the intermediate region and gradually propagates outward. It is noteworthy that most of the cracks propagate outward at a specific angle, which is macroscopically roughly in line with the rupture angle defined in the Mohr–Coulomb Criterion.

**Figure 11:**
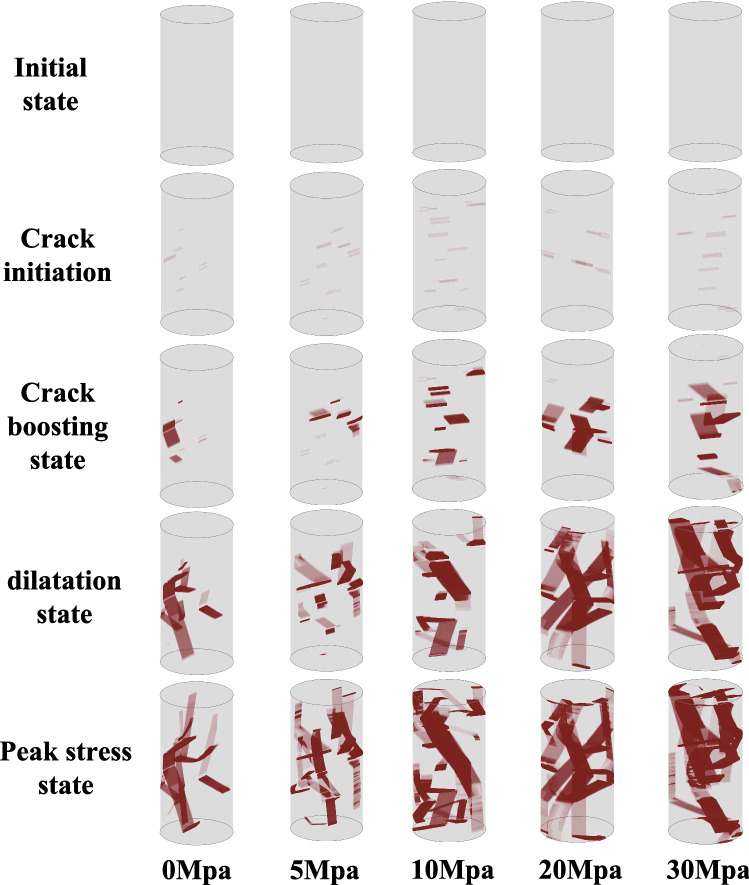
Simulation of the fracture behavior under different confining stress.

To visualize the fracture behavior, Fig. 11 shows the simulation results for each state under different confining stresses. It is obvious that the cracks inside the rock specimens tend to grow and form a particular pattern. At uniaxial loading state, the fracture plane is relatively aligned with the axial stress direction, while larger confining stresses will lead to a more inclined failure plane as indicated by the Mohr–Coulomb criterion. We can also spot that larger confining stress may also lead to a more complex fracture pattern along with the main failure plane. This is reasonable because at lower confining stresses, the axial strength of the rock specimen is not sufficient to support the formation of a complex crack pattern, and failure occurs more quickly when the crack reaches the edge of the specimen. However, higher confining stresses can inhibit spalling or shedding when the cracks reach the edges, so the rock specimen will retain its original shape and gain more strength to form relatively complex fracture patterns with crushing cracks at the edges. It is notable that only a very small number of inherited cracks will initiate and propagate during fracturing, which is also supported by the observations in past experiments^42,43^. However, due to mathematical constraints, our model cannot present the genuine state of real rock failure and may produce some undesirable results.

**Figure 12:**
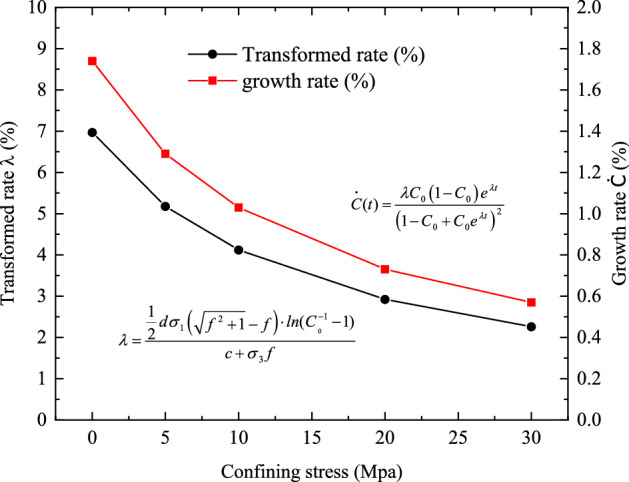
Transformation rate and growth rate over confining stress.

According to the simulation results in Fig. 11, the crack length increases with increasing confining stress, which is commonsensible because greater confining stresses lead to larger axial stresses, resulting in higher fracture energies and ultimately higher fracture increments as indicated in Eq. (35). It seems that the cracks are gradually dominating the rock samples. However, Fig. 12 shows the variation of transformation rate and growth rate over different confining stress, which indicates that the overall growth rate of fractures is much lower than expected and the growth rate decreases with increasing confining stress. This phenomenon is aligned with the findings in Refs.^42,43^. The growth rate in our model represents the probability that those fractured voxels can propagate when the initial fracture stress exceeds the cohesion. Specifically, for example, the probability of the initial cracks in a rock sample under 30 Mpa confining stress is around 0.6%, which means that around 6 out of 1000 sets of cracked voxels in this granite sample are able to propagate during the entire course of the compression test. Therefore, the crack pattern formed in Fig. 11 is contributed by a small number of initial cracks, and most of the cracks inside the rock sample will not be triggered and enlarged. This phenomenon can be explained by the fact that cracks tend to propagate and coalesce along the weakest parts, so once some initial cracks start to grow in a small area, cracks around them are likely to follow, while other cracks away from this area are less likely to be triggered. This is also supported by our direction assumption in “The growth direction of cracks”, where the attractive direction (see Eq. (16)) dominates the crack orientation to some extent by clustering the cracks together.

### Analysis of key parameters

**Figure 13:**
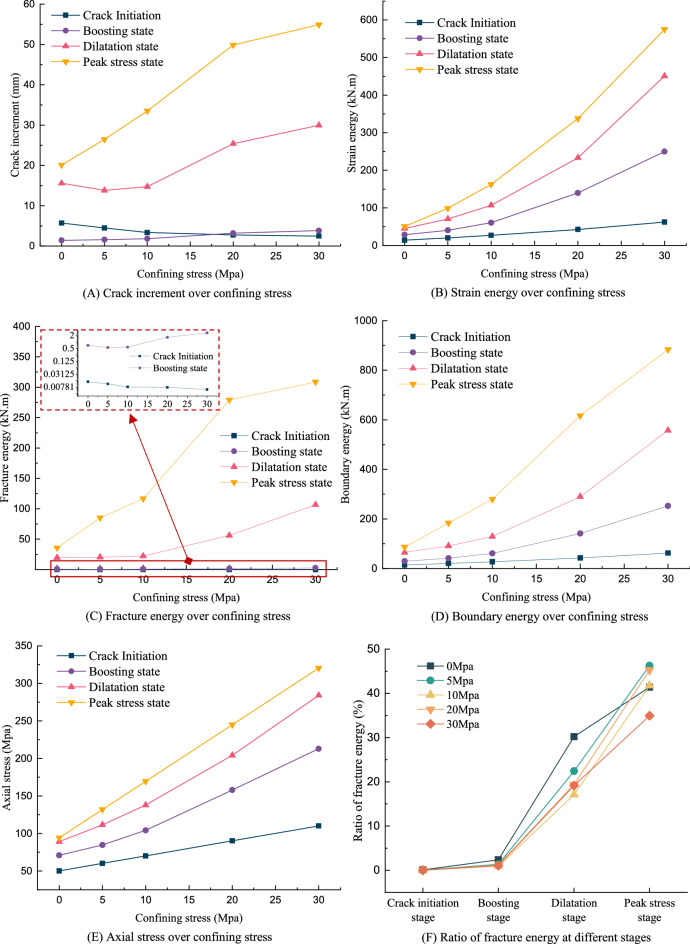
Comparison of key parameters over different confining stress.

Figure 13 presents the simulation results of several key parameters at different compression stages under different confining stress. It can be seen from Fig. 13E that the rock samples are prone to fail after the dilatation state (or crack damage point) at lower confining stresses. Furthermore, it can be seen that the pink triangles (stress at dilatation state) are much closer to the yellow triangles (peak stress) under lower confining stress (damage stress is about 81.7% of the peak stress at 5 Mpa), which means that rock samples (especially granites) are more prone to failure after the dilatation stage under lower confining stress. therefore, once the lateral expansion rate exceeds the axial compression rate, the rock will be damaged quickly due to the low lateral confinement. This result is also supported by Ref.^44^ that the dilatation state may be closed enough to the peak stress state or not exist when the confining stress is relatively small on brittle materials. According to Fig. 13A, we can also observe that the crack increment at the crack initiation stage decreases as the confining stress increases. However, larger confining stress increases the crack increment when the axial stress keeps increasing. This contradictory phenomenon can be reasonably explained to some extent by our crack growth model. As mentioned in “Methodology”, the crack increment is indirectly influenced by crack growth rate $C^{\prime}(t)$ in Eq. (3), which is mainly contributed by the transformation rate $\lambda$ in Eq. (11). As indicated in Eq. (11), as the confining stress $\sigma_{2}$ increases, the transformation rate $\lambda$ will decrease, which leads to an initial decrease in the crack increment. However, the crack increment is also strongly affected by the fracture energy $E_{f}^{t}$ in Eq. (25) which dominates the crack extension amount. This means that increasing axial stress will produce a higher fracture energy and dominate the crack growth in the subsequent stages, especially at higher confining stresses. Therefore, the simulation reveals a complex interconnection between the transformation rate and fracture energy, that is, the transformation rate dominates the crack increment in the beginning while the fracture energy controls the crack increment as the increase of the axial stress.

By observing the plot of the strain energy, the fracture energy and the boundary energy, we can also conclude that with the increasing of the confining stress, energy parameters will grow exponentially as the axial stress increases. It is worth noting that the strain energy predominates at lower axial stresses, while the fracture energy will gradually enlarge its ratio when the axial stress increases. This phenomenon reflects the essence of the crack growth model in Eq. (1) that crack growth behavior follows the pattern of logistic growth behavior. In details, we can observe from Fig. 13F that the fracture energy only takes over a very small amount of the overall energy before Boosting stage while strain energy is dominant due to less fractured voxels proportion at the early stages. After the boosting stage, fractured energy will grow exponentially due to increasing proportion of fractured voxels. But in overall, strain energy always occupies the largest energy portion.

## Discussion

According to the simulation results, the compression test can be divided into four stages, namely, the elastic stage, crack initiation stage, stable growing stage and unstable growth stage.

At the beginning of the crack initiation stage, a significant crack increment surge signal occurs due to the sudden release of the elastic energy accumulated in the first stage. Crack initiation will not happen until the axial stress reaches the initial fracture stress defined in Eq. (32). According to the definition of the initial fracture stress, by letting the initial fracture stress equals to the cohesion, the initial fracture stress of the $kth$ crack can be expressed as:

39 $$\sigma_{k}^{t} = \frac{{2\mu c + \sigma_{2} \cdot \left( {\sin 2\theta_{k}^{t} - \mu \cos 2\theta_{k}^{t} + \mu } \right)}}{{\sin 2\theta_{k}^{t} - \mu \cos 2\theta_{k}^{t} - \mu }}.$$

Equation (39) is good for knowing when the $kth$ crack will initiate but not applicable for knowing when the rock sample starts to fracture. Thus, a simple modification to (39) is to let $\theta_{k}^{t}$ equals to the rupture angle $\theta$, and in this manner, the initial fracture stress can be modified as:

40 $$\sigma_{i}^{t} = \frac{{2\mu c + \sigma_{2} \cdot \left( {\sin 2\theta - \mu \cos 2\theta + \mu } \right)}}{\sin 2\theta - \mu \cos 2\theta - \mu }.$$

**Figure 14:**
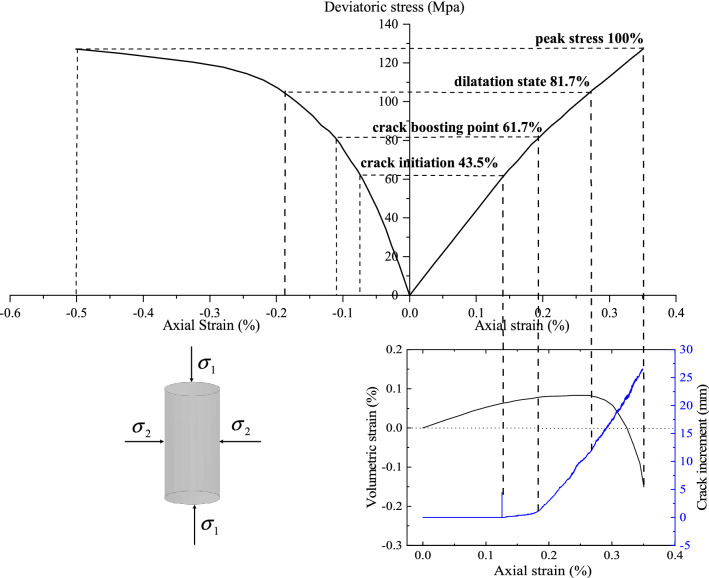
Stress–strain curves under 5 Mpa confining stress showing the stages of crack growth.

In this paper, the initial fracture stress is around 43.5% of the peak stress (see Fig. 14). To verify whether Eq. (40) is applicable to calculate the initial fracture stress, we can divide Eq. (40) by (36) and obtain the crack initiation ratio:

41 $$R_{i} = \frac{{2\mu c + \sigma_{2} \cdot \left( {\sin 2\theta - \mu \cos 2\theta + \mu } \right)}}{{\left( {\sin 2\theta - \mu \cos 2\theta - \mu } \right) \cdot \left[ {\left( {\sqrt {f^{2} + 1} + f} \right)^{2} \cdot \sigma_{2} + \left( {\sqrt {f^{2} + 1} + f} \right) \cdot 2c} \right]}}.$$

By substituting the list of parameters in Table 3 into (41), we can obtain $R_{i} { = }43.91\%$ which is fairly close to our simulation result (43.5%) in Fig. 14. Therefore, Eq. (41) is able to calculate the initial fracture stress and thus know when the rock sample will start to crack.

**Table 3.** Mechanical parameters used in our model.

Parameter	Symbol	Quantity	Unit
Confining stress	$\sigma_{2}$	5	Mpa
Internal friction angle	$\phi$	49.96	°
Cohesion	$c$	17.19	Mpa
Coefficient of friction	$\mu$	0.6	–

The derivative of the volumetric strain will approach 0 before entering the stable growth stage, which tells us that the circumferential growth rate of the rock specimen exceeds the axial growth rate. This is critical because after this point, the crack increment rate will accelerate exponentially, leading to coalescence of microcracks.

The compression test enters the unstable growth stage when the rock sample reaches the dilatation point (or the crack damage point) where the volumetric strain of the rock sample becomes 0. The crack increment keeps boosting and accelerating without a sign of stop and finally ends up with failure at the peak stress point, leading to macro-cracks inside the rock samples gradually propagating and penetrating to the edges of the rock sample. It is noticeable that the axial stress at the dilatation state is around 80% of the peak stress, which can also be calculated by letting $\varepsilon_{v} + \varepsilon_{pv} = 2\left( {\varepsilon_{h} + \varepsilon_{ph} } \right)$ with Eqs. (23) and (31). Thus, the axial stress at the dilatation state is derived as:

42 $$\sigma_{d} = \frac{{\frac{{EV\left( {C_{d} - C_{0} } \right)\left( {2H - Rtan\theta } \right)}}{{4\pi R^{3} Htan\theta }} + 2\sigma_{2} }}{1 + 2\nu },$$

where $C_{d}$ is the volume ratio of the cracks at the dilatation state. It is worth noting that Eq. (42) has no upper limit, thus when Eq. (42) is larger than the peak stress, the dilatation state will not exist. This is accepted by many research works that the dilatation state does not always exist^36,37^.

## Conclusion and future work

To conclude, the proposed crack growth model in this paper explains the fracture behavior and the transformation rules of cracks from the view of natural colony growth behavior, which is expounded and verified by theoretical deduction, experimental tests and simulation verification. This may provide some novel views or enlightenments to the research of crack evolution in the following points:

1. This paper considers crack voxels of rocks under compression as individuals who can initiate, propagate and coalesce by following the colony growth behavior of nature. Based on this assumption, we proposed a dynamic growth model combined with Mohr–Coulomb criterion to reveal this fracture behavior.
2. Our model combined the maximum shear stress criterion (Mohr–Coulomb) and energy criterion together. The maximum principal shear stress criterion controls the initiation of cracks (see Eq. (32) and the failure of rocks (see Eqs. (6)–(10)), and the energy criterion controls the speed of crack propagation (see Eqs. (33)–(35)).
3. Based on the assumption of natural colony growth, we introduce four propagation directions of cracks to simulate the genuine state of fracture pattern of rock under compression. Simulation results show that the failure plane under lower confining stress is relatively aligned with the axial stress path while larger confining stress may generate a more complex but inclined failure plane.
4. Compared with the experimental text results and the simulation results of PFC3D, our model can provide a more genuine failure pattern in different confining stress. Furthermore, our model outperforms PFC3D in the way that it can better present the fracture pattern at lower confining stresses.

However, the proposed method still contains several limitations to be improved in the future. The proposed model only characterizes the fracture behavior before the peak stress state, the after-peak state is still hard to be characterized. Furthermore, crack closure phenomenon is neglected in this paper because it involves complicated game behaviors between cracks and non-fractured parts, which cannot be well explained in this crack growth model. Additionally, only shearing failure mode is considered in our simulation model, which can only characterize high stress rock failure to some extent. Finally, the crack growth model is only verified in the porous rocks with tiny inherited cracks in this paper, that is, the proposed model may not be applicable for those rock masses with distinct flaws or discontinuities. Future research will focus on the above limitations and provide insightful analysis of them.

## Data Availability

The datasets generated during and/or analyzed during the current study are available from the corresponding author on reasonable request.

## List of symbols

$C$: The volumetric ratio of fractured voxels to the volume of the rock body
$I$: The volumetric ratio of the non-fractured part (or the skeleton)
$\lambda$: Transformation rate between the volumetric ratio of fractured voxels and the volumetric ratio of the rock skeleton
$$C_{0}$$: Initial porosity
$t_{m}$: Peak stress moment
$\sigma_{1}$, $\sigma_{2}$: The vertical and lateral (confining) stress
$c$: The cohesion stress
$\phi$: The internal friction angle of the rocks
$\theta$: The rupture angle based on Mohr–Coulomb criterion
$\tau$: The shear stress
$\sigma$: The principal stress
$f$: The friction coefficient
$d\sigma_{1}$: The loading rate in the compression tests
$\overrightarrow {{V_{k}^{t} }}$: The growth direction of cracks
$\overrightarrow {{V_{{_{ik} }}^{t} }}$: The inertial direction
$\overrightarrow {{V_{{_{ak} }}^{t} }}$: The attractive direction
$\overrightarrow {{V_{{_{mk} }}^{t} }}$: The Coulomb direction
$\overrightarrow {{V_{{_{ek} }}^{t} }}$: The edge attraction direction
$\alpha_{{_{1k} }}^{t}$: The inertial weight
$l_{{_{k} }}^{t}$: The length of the $kth$ crack at time $t$
$p_{k}^{t}$: The current crack tips
$\omega_{j}^{t}$: The attracting strength
$s_{j}^{k}$: The attractive radius
$\vartheta$: The attenuation factor which ranges from 0 to 1
$\alpha_{{_{2k} }}^{t}$: The attracting weight
$u_{{_{k} }}^{t}$: The magnitude of the sum of vectors for which the current crack tips $p_{k}^{t}$ points to all other crack tips $p_{j}^{t}$ within the neighborhood $\mathop U\limits^{ \circ } (p_{k}^{t} ,\delta )$
$\xi_{{_{k} }}^{t}$: The angle between the inertial direction of the crack and the vertical stress
$\alpha_{{_{3k} }}^{t}$: The Coulomb weight
$D_{ku}^{t}$, $D_{kb}^{t}$: The distance of the $kth$ crack tip to the up and the bottom edge of the specimen respectively at time
$\alpha_{{_{4k} }}^{t}$: The edge weight
$H$, $R$: The height and the radius of the rock specimen
$E_{all}^{t}$: The energy absorbed by the rock sample at time $t$
$\sigma_{{_{x} }}^{t}$, $\sigma_{{_{y} }}^{t}$ and $\sigma_{{_{z} }}^{t}$: The stress in triaxial directions at time $t$
$\varepsilon_{{_{x} }}^{t}$, $\varepsilon_{{_{y} }}^{t}$ and $\varepsilon_{{_{z} }}^{t}$: The triaxial elastic strain at time $t$
$E_{e}^{t}$: The elastic energy
$E_{f}^{t}$: The fracture energy
$V$: The volume of the rock specimen
$E$, $\nu$: The elastic modules and the Possion’s ratio of the rock sample
$r$: The thickness of the inherited microcracks
$w$: The width of the inherited microcracks
$\mu$: The coefficient of friction which is defined in ^1^
$\sigma_{{_{k} }}^{{t{ - }1}}$: The initial fracture stress
$\tau_{kr}$, $p_{k}$: The resolved shear stress and the normal stress on the face of crack *k*
$\theta_{k}$: The orientation angle between the crack $k$ and the horizontal axis
$\delta l_{k}^{t}$: Crack increment of the *kth* crack at time $t$
$E_{p}^{t}$: The plastic energy
$\varepsilon_{{_{px} }}^{t}$, $\varepsilon_{{_{py} }}^{t}$ and $\varepsilon_{{_{pz} }}^{t}$: The triaxial plastic strain at time $t$
$\delta {\mathcal{V}}^{{t{ - }1}}$: The volume increment of the cracks
$l$: The length of the equivalent elliptical thin plane
$\ell$: The thickness of the equivalent elliptical thin plane
$\delta v$, $\delta h$: The equivalent vertical and lateral increment caused by the crack increment
$\varepsilon_{pv}$, $\varepsilon_{ph}$: The vertical and lateral plastic strain of the rock sample
$E_{k}^{t}$: The fracture energy of the $kth$ crack at time $t$
$R_{k}^{t}$: The unit resistant energy of the $kth$ crack at time $t$
$\sigma_{m}$: The peak axial strength of the rock sample
$p$: The slope of strength envelops over confining stress
$q$: The uniaxial strength
$\sigma_{i}^{t}$: The modified initial fracture stress
$R_{i}$: The ratio of the initial fracture stress to the peak stress
$\sigma_{d}$: The axial stress at the dilatation state
$C_{d}$: The volume ratio of the cracks at the dilatation state
